# The Impact of SARS-CoV-2 (COVID-19) and its Lockdown Measures on the Mental and Functional Health of Older Individuals

**DOI:** 10.1007/s11126-021-09943-6

**Published:** 2021-08-20

**Authors:** Maria Chiara Fastame, Ilaria Mulas, Valeria Putzu, Gesuina Asoni, Daniela Viale, Irene Mameli, Massimiliano Pau

**Affiliations:** 1 Department of Pedagogy, Psychology, Philosophy, University of Cagliari, Via Is Mirrionis 1, Cagliari, Italy; 2 Department of Mechanical, Chemical and Materials Engineering, University of Cagliari, Piazza D’Armi, 09123 Cagliari, Italy; 3 Center for Cognitive Disorders and Dementia, Geriatric Unit SS. Trinità Hospital, Cagliari, Italy

**Keywords:** COVID-19, Psychological well-being, Gait analysis, Motor efficiency, Aging

## Abstract

The effect of the COVID-19 on the physical and mental health of Italian older individuals displaying signs of cognitive deterioration has not been deeply investigated. This longitudinal study examined the impact of COVID-19 lockdown measures on the psychological well-being and motor efficiency of a sample of Italian community-dwellers with and without cognitive decline. Forty-seven participants underwent instrumental gait analysis performed in ecological setting using wearable sensors, and completed a battery of tasks assessing cognitive functioning and psychological well-being, before and after the full lockdown due to the COVID-19 spreading. A series of Multivariate Analyses of Variance (MANOVAs) documented that the superior gait performance of the cognitively healthy participants exhibited before the COVID-19 spread, vanished when they were tested at the end of the lockdown period. Moreover, before the outbreak of the COVID-19, cognitively healthy participants and those with signs of cognitive decline reported similar levels of psychological well-being, whereas, after the lockdown, the former group reported better coping, emotional competencies, and general well-being than the participants displaying signs of cognitive decline. In conclusion, the full COVID-19 outbreak had a significant impact on the mental and motor functioning of older individuals with and without signs of cognitive deterioration living in Italy.

## Introduction

Due to the tremendous impact of the COVID-19 outbreak (a viral respiratory dis-ease that originated from the coronavirus SARS-CoV-2) that first appeared in Italy in February 2020, on March 9th, 2020 the Italian government imposed a nationwide quarantine, which included the impossibility for any citizen to stay out of home, except for specific reasons (i.e. work, health and shopping for essential goods and medications). During such lockdown, which remained effective for 68 days, citizens were prevented from performing any kind of either structured or free physical activity. During the lockdown period, the only possible form of allowed movement was a single daily short walk at a distance not exceeding 200 m from the place of residence. Moreover, any sort of social contact even with other family members living elsewhere was not allowed in order to avoid the spread of the coronavirus.

Although it was essential to counteract the dramatic consequences of the out-break, such measure immediately raised many concerns especially for specific population groups such as older adults, for which the inactive lifestyle is seriously detrimental to physical and cognitive health and might even accentuate some anomalous motor and psychological patterns usually exhibited in late adulthood [1]. In this regard, it is known that in older adults prolonged forced inactivity may be associated with several adverse health outcomes, such as exacerbation of age-related muscle wasting, accelerated progression of sarcopenia, development of comorbidities, and increased risk of fall, which may result in consequent fractures and disabilities [2, 3]. Therefore, the direct consequence is that social isolation due to the pandemic outbreak might exacerbate the physiologic decline of typical mobility documented in older individuals [4]. Moreover, a recent study reported the negative influences of the lockdown measures on mental health (e.g., high rates of negative mood) in the Italian population [5]. However, relatively to the adult lifespan, it has been documented that older individuals reported fewer COVID-19-related worries and fewer anxious and stress-related symptoms than younger participants [5–7]. Following this, Rossi et al. [7] argued that when the effects of some confounding factors (i.e., region of residence, education, and gender) were controlled for, the better mental health outcomes observed in the Italian older adults (i.e., people aged 60 and over) could be due to the fact that they use better resilience to face the adversities and stressful events (such as the occurring COVID-19 pandemic) compared with younger individuals. Despite this, Tyrrell and Williams [8] highlighted the risk of the onset of psychological mental health complications due to the COVID-19 social distancing (i.e., loneliness and social isolation) in late adulthood.

However, to our knowledge, the impact of the COVID-19 lockdown measures on the self-reported mental health and motor functioning of Italian community-based older people has not been concurrently explored, especially in those individuals displaying signs of cognitive deterioration. This is very relevant because if cognitive decline poses a threat to the quality of life of older people, its impact could be even more detrimental due to the occurrence of the lockdown measures associated with the COVID-19 spread.

### Purpose of the Study

This study was aimed at examining the impact of the COVID-19 lockdown measures on the psychological well-being and motor efficiency of community-dwelling Italian older individuals with and without signs of cognitive decline. In particular, we selected gait as a basic motor task strongly associated with physical fitness in older adults [9] and recognized as capable to influence a wide range of activities of daily living, other than being implicated in the risk of falls, hospitalization, and death [10]. Following Tyrrell and Williams [8] the negative impact of lockdown measures on perceived well-being was hypothesized. However, in line with further evidence [6, 7] no significant differences before and after the introduction of lockdown measures due to the COVID-19 spread could be expected in terms of perceived mental health reported by the older participants. Finally, a decremental effect of motor efficiency was expected after the end of the COVID-19 lockdown, especially among participants dis-playing signs of cognitive decline [2, 3].

## Method

### Participants

Forty-seven individuals aged 66-89 years (Mage = 75.7 years, SD = 5.7 years), 17 males and 30 females, were recruited at the Center for Cognitive Disorders and Dementia (Geriatric Unit, SS. Trinità Hospital, Cagliari, Italy) and took voluntarily part in the study. Two main inclusion criteria had to be met to be enrolled in the study: 1) being community-based; 2) being free from neurologic disorders interfering with mobility (e.g. Parkinson’s disease, multiple sclerosis, and stroke), severe symptomatic orthopedic conditions, and, in general, inability to walk independently. Based on the cut-off indices provided for the Italian validation [11] of Addenbrooke’s Cognitive Examination-Revised (ACE-R) [12], participants were assigned to the cognitively healthy group or the group of individuals exhibiting signs of cognitive deterioration (i.e., cognitively impaired group), respectively. Gender (χ2 = 3, df = 1, p = .083) and educational attainment (χ2 = 2.08, df = 1, p = .149) which was dichotomized as low (i.e., ≤ 8 years, n = 28) and high (i.e., > 8 years, n = 19) were equally distributed among participants. Table 1 summarizes the socio-demographic characteristics of the sample and scores on two screening tests assessing global cognitive functioning, namely the ACE-R and Mini-Mental State Examination (MMSE) [12, 13].

**Table 1:** Sociodemographic characteristics, BMI, and global cognitive efficiency (i.e., MMSE and ACE-R scores) of cognitively healthy participants and those showing signs of cognitive decline (i.e., Cog-nitively Impaired Group). Information was recorded before the COVID-19 outbreak in Sardinia (Italy), where the participants were recruited (i.e., between December 2019 and March 5, 2020). Standard deviations are reported in parentheses

**Variable**	**Cognitively Healthy** **Group**	**Cognitively** **Impaired** **Group**	**χ2**	**t**	**df**	**p**
n Gender males females	21 9 12	26 8 18	.532 .735		1 1	.466 .543
Age (years)	M = 76.2 (SD = 5.8)	M = 75.7 (SD = 5.5)		.352	45	.727
BMI MMSE score ACE-R	M = 24.91 (SD = 3.8) M = 27.6 (SD = 2.6) M = 81.38 (SD = 12.8)	M = 23.5 (SD = 3.3) M = 22.2 (SD = 4.2) M = 56.31 (SD = 14.5)		1.38 5.06 6.21	45 45 45	.175 < .005 < .005

As reported in this table, no differences were found in terms of age and Body Mass Index (BMI) between cognitively healthy participants and those showing signs of cognitive deterioration.

### Materials

Each respondent completed the following tools:

The Mini-Mental State Examination (MMSE) [13] was used to screen participants’ global cognitive functioning. This tool is composed of 18 items assessing different cognitive processes such as attention, spatiotemporal orientation, motor coordination, short and long-term memory. A score < 24/30 indicated the occurrence of suspected signs of cognitive impairment;

The Addenbrooke’s Cognitive Examination Revised (ACE-R) [12, Italian validation: 11] is a pencil-and-paper battery encompassing 5 subtests designed to assess the efficiency of attention, memory, verbal fluency, language (e.g., comprehension), and visuo-spatial skills (maximum score = 100). A score ≤ 79 for people aged ≤ 75 years and a score ≤ 60 for participants over 75 years were the cut-off indices used to detect participants with signs of cognitive deterioration [11];

The Psychological Well-Being and Aging Questionnaire [PWBAQ, 14] is a 37-item inventory designed to assess general psychological well-being, coping strategies (i.e., ability to solve daily problems), emotional competencies (i.e., ability to understand and share one’s emotional state with others) and personal satisfaction (i.e., satisfaction with one’s past, present and future life events), respectively. For each statement, the participant had to assess on a 4-point Likert scale the frequency (1 = never vs. 4 = al-ways) in which that situation occurred in his/her daily life (maximum total score = 148). The authors reported that in cognitively healthy older individuals, a total score ≥ 115 indicates the highest level of general psychological well-being, whereas a score ≤103 indicates a low level of general psychological well-being. According to De Beni et al. [14], this measure has good internal consistency (Cronbach’s alpha = .81).

Gait was objectively assessed using a miniaturized wearable inertial sensor (G-Sensor®, BTS Bioengineering S.p.A., Italy) previously employed for similar investigations in older adults [15] and which was attached to the individual’s trunk at S1 vertebrae location. Participants were requested to walk along a 30-m hallway, following a straight trajectory at a self-selected speed and in the most natural manner. The accelerations recorded by the device were transmitted in real-time to a notebook and then processed to calculate the main spatiotemporal parameters of gait. Specifically, for the aims of the current study, the following parameters were detected: gait speed (i.e., the mean velocity of progression, expressed in m s ^– 1^), and stride length (i.e., the longitudinal distance between two successive ground contacts of the same foot, expressed in meters).

### Procedure

After completion of written informed consent, each participant was first tested in the period between December 2019 and March 5, 2020 (i.e., before the spreading of the COVID-19 in Italy), and then he/she was retested in the period included between July 13, 2020, and August 4, 2020, that is, when the Center for Cognitive Disorders and Dementia was allowed to resume regular clinical activity, after the so-called first wave of the COVID-19 spread in Italy. In each experimental session, first, each participant was individually presented with the MMSE, then the ACE-R was administered and then the psychological well-being questionnaire was completed. To reduce the fatigue effect, the examiner read each item aloud and when it was necessary (e.g., the date, the location where the testing occurred) she wrote the answers provided by the participant on the response sheet. Finally, some anthropometric features (i.e. stature, body mass, and BMI) and the gait parameters were assessed. Overall, each experimental session lasted approximately 60 minutes.

## Results

A multivariate analysis of variance (MANOVA) was conducted to analyze the impact of cognitive deterioration (i.e., cognitively healthy group vs. cognitively im-paired one) on the PWBAQ indexes before the COVID-19 outbreak. The Multivariate tests did not document the significant main effect of the cognitive deterioration [Wilks’ λ = .926, df = 4;42, p = .536]. Overall, at baseline, the two groups reported similar scores in the general well-being [F(1,45)= 2.08, p = .16], coping [F(1,45)= 1.45, p = .23], person-al satisfaction [F(1,45)= .815, p = .37], and emotional competencies [F(1,45)= 2.86, p = .10] conditions. Table 2 illustrates the effect of cognitive decline on each PWBAQ measure.

**Table 2:** Means (i.e., M), Standard Deviations (i.e., SD), and Multivariate Analysis of Variance displaying the effect of cognitive decline on total Psychological Well-Being (i.e., PWBAQ-tot), coping strategies (i.e., PWBAQ-cop), personal satisfaction (i.e., PWBAQ-ps), and emotional competencies (i.e., PWBAQ-ec) indices reported before the COVID-19 outbreak (in the period between December 2019 and March 5, 2020)

Measure	Cognitively Healthy Group		Cognitively Impaired Group		*F*(1, 45)	p	η^2^
	*M*	*SD*	*M*	*SD*			
PWBAQ-tot	113.35	16.78	103.40	26.90	2.08	.16	
PWBAQ-cop	24.45	5.63	21.96	7.74	1.45	.23	
PWBAQ-ps	35.45	5.67	33.47	8.38	.815	.37	
PWBAQ-ec	31.25	4.90	28.64	5.31	2.86	.10	

Then a further MANOVA was conducted to examine the impact of cognitive de-terioration on the PWBAQ indexes recorded after the end of the COVID-19 lockdown measures. The Multivariate tests revealed the significant main effect of the cognitive deterioration [Wilks’ λ = .602, df = 4;42, p < .005]. At follow-up, cognitively healthy participants reported greater general well-being [F(1,45)= 4.38, p = .04, η2 = .09], coping [F(1,45)= 13.57, p = .001, η2 = .23], and emotional competencies [F(1,45)= 9.40, p = .004, η2 = .17] than the group exhibiting signs of cognitive decline. In contrast, no differences between the groups were found in the personal satisfaction condition [F(1,45)= .802, p = .37]. Table 3 summarizes the effect of cognitive decline on each PWBAQ measure af-ter the COVID-19 first wave.

**Table 3:** Means (i.e., M), Standard Deviations (i.e., SD), and Analyses of Variance in total Psychological Well-Being Aging Questionnaire (i.e., PWBAQ-tot), coping strategies (i.e., PWBAQ-cop), personal satisfaction (i.e., PWBAQ-ps), and emotional competences (i.e., PWBAQ-ec) indexes reported after the introduction of the COVID-19 lockdown measures (July-August 2020)

Measure	Cognitively Healthy Group		Cognitively Impaired Group		*F*(1, 45)	p	η^2^
	*M*	*SD*	*M*	*SD*			
PWBAQ-tot	113.62	16.35	103.15	17.56	4.38	.04	.09
PWBAQ-cop	26.05	4.82	20.61	5.18	13.57	.001	.23
PWBAQ-ps	34.05	5.07	32.57	6	.802	.37	
PWBAQ-ec	32.57	4.32	28.46	4.76	9.40	.004	.17

The above-mentioned analyses were replicated to investigate the effect of cognitive decline on some motor measures recorded before and after the COVID-19 out-break that occurred in winter 2020, respectively. Multivariate tests revealed the significant main effect of cognitive deterioration on walking speed and stride length be-fore the pandemic [Wilks’ λ = .823, df = 2;44, p = .016] but not after the introduction of the associated social distance [Wilks’ λ = .943, df = 2;44, p = .276]. Indeed, at baseline cognitively healthy participants outperformed the group with signs of cognitive decline in terms of gait speed [F(1,45)= 5.84, p = .02, η2 = .11] and stride length [F(1,45)= 8.64, p = .005, η2 = .16]. In contrast, after the first wave of the COVID-19 lockdown, no differences were found between the groups in terms of gait speed [F(1,45)= 2.45, p = .12] and stride length [F(1,45)= 2.69, p = .11] parameters. Table 4 illustrates the impact of cognitive decline on each motor index that was recorded before the COVID-19 outbreak and after the end of the lockdown measures.

**Table 4:** Means (i.e., M), Standard Deviations (i.e., SD), and Multivariate Analyses of Variance showing the impact of cognitive decline on gait speed and stride length of cognitively healthy and cognitively impaired participants. The motor parameters were recorded before the pandemic (i.e., pre-COVID19) and after the end of the nationwide lockdown restrictions (i.e., post-COVID19)

Measure	Cognitively Healthy Group		Cognitively Impaired Group		*F*(1, 45)	p	η^2^
	*M*	*SD*	*M*	*SD*			
Gait speed pre-COVID19	.95	.23	.77	.27	5.84	.02	.11
Stride Length pre-COVID19	1.08	.22	.87	.27	8.64	.005	.16
Gait speed post-COVID19	.87	.32	.74	.28	2.45	.12	
Stride Length post-COVID19	.99	.33	.83	.31	2.69	.11	

Moreover, two paired-sample t·tests were conducted to evaluate the impact of the COVID-19 lockdown measures on cognitively healthy participants’ gait measures recorded at baseline and after the end of the lockdown restrictions. There was a statistically significant decrease in cognitively intact participants’ stride length from baseline to follow-up assessment [t(20) = 2.22, p = .038]. The eta squared statistic (.49) indicated a large effect size. Moreover, the impact of the COVID-19 lockdown measures approached significance in the speed condition [t(20) = 1.93, p = .06]. Finally, when the same analyses were replicated for the cognitively impaired group, the effect of the COVID-19 restrictions was not significant in the walking speed [t(25) = .759, p = .45] and stride length [t(25) = .689, p = .50] conditions.

## Discussion

This is the first longitudinal study examining the impact of the COVID-19 lockdown measures on perceived psychological well-being and objectively assessed and concurrently recorded motor efficiency in a sample of Italian older individuals with and without signs of cognitive deterioration. Indeed, to the best of our knowledge, at present, no studies have been conducted to pursue this goal on typically and atypically developing older adults.

Overall, the current outcomes extend previous findings and provide empirical support for the negative impact of social distancing due to the introduction of the lockdown measures on both motor functioning [2, 3] and perceived mental well-being [8] in late adulthood. Indeed, with respect to motor performance, these results documented that before the COVID-19 outbreak, our cognitively healthy participants reported a more performant gait (i.e., higher speed and longer stride length) than peers with signs of cognitive decline. The differences in locomotion abilities of the participants with signs of cognitive decline detected at baseline are consistent with some previous findings showing that gait performance of older individuals are linked to their cognitive skills [16–18]. However, the current results also highlighted that the asymmetry in mobility performance that was found comparing the cognitively intact group with the cognitively impaired one at baseline was lost at follow-up, that is when outdoor motor activities were restricted due to the introduction of the COVID-19 lockdown measures. Therefore, extending the outcomes of a study recently performed on a larger sample of Italian adults [15], we found an overall worsening of our participant's gait performance, which can be considered an evident result of their physical inactivity [19] due to the lockdown restrictions. Indeed, during the nationwide quarantine in the winter-spring 2020, the Italian government established that citizens were prevented from performing any kind of physical activity, that is, for 68 days Italians limited their motricity to a short daily walk within 200 meters of distance from their home. However, it must be noticed that the loss of motor skills at follow-up did not involve those participants with signs of cognitive impairment, probably since their levels of physical activity were already low before the lockdown [20], as revealed by their worst gait capabilities at baseline. In contrast, our data indicate that the lockdown prescription particularly affected the movement skills of the typically developing older group (i.e., with an evident decrease in the stride length at follow-up), since they could not be physically active for more than two months.

Additionally, current outcomes suggest that the occurrence of the pandemic negatively impacted the mental health of older individuals. Indeed, before the COVID-19 out-break, our participants with and without signs of cognitive decline self-reported a similar level of psychological well-being. That is, despite that at baseline the cognitively healthy participants reported medium-high levels of general psychological well-being, and the group with signs of cognitive decline reported low psychological well-being, no statistically significant differences were found between the two groups. Thus, in line with previous findings [21], one can speculate that having an active lifestyle and being socially connected with family members can enhance the perceived mental health in late adulthood. Furthermore, these findings also showed that after the introduction of the lockdown measures, the cognitively healthy group outperformed the cognitively deteriorated one in terms of coping, emotional competencies, and total psychological well-being. From a psychological viewpoint, these outcomes highlighted that the COVID-19 restrictions particularly affected the cognitively weakest participants which suffered particularly for the social distancing (i.e., significant reduces emotional competencies) and expressed more difficulties in dealing with a stressful event like the pandemic (i.e., at follow-up they reported fewer coping strategies than the cognitively healthy group). Therefore, it is plausible to hypothesize that the impossibility to carry on with their routines in daily life, without the support of other family members, let the participants with signs of cognitive decline feel less competent in the management of their routines and in tackling the problems related to the COVID-19 spread. In contrast, the cognitively healthy participants reported feeling confident in facing the difficulties related to the pandemic outbreak and despite the social distancing, they felt emotionally connected with people they cared for. Therefore, extending previous findings [8], current outcomes suggest that the social distancing imposed by the COVID-19 restrictions (included those with family members living elsewhere) has determined a mental health asymmetry between the older individuals with and without signs of cognitive decline. This conclusion is consistent with some very recent findings highlighting the negative consequences of the COVID-19 pandemic on the social well-being (i.e., greater loneliness) and mental health (i.e., increased occurrence of depressive symptoms) of a sample of American older individuals [22].

Some limitations of the study should be acknowledged. Firstly, given the lack of further follow-up data, we are unable to assess whether the modifications here observed in both psychological and gait measures were (fully or partly) reversible. Secondly, since the battery of tests proposed in the current study is limited, future research should use additional tools to examine, for instance, the impact of the COVID-19 spread on the efficiency of further psychological processes such as the executive functions (i.e., which in the current study were assessed only through the verbal fluency subtest of the ACE-R) and their relationship with the motor functioning of the older community-dwellers. This is quite relevant since a body of evidence shows that the deficits in the executive functions’ tasks are associated with the development of motor decline and reduced mobility in late adulthood [23]. Moreover, following Rossi et al. [7] a further limitation of this study is that it does not disentangle the role played by resilience on the perceived mental health of the participants (i.e., especially those exhibiting signs of cognitive decline) before and after the introduction of the COVID-19 lockdown measures. Finally, considering the limited sample size, caution is needed in generalizing these findings, especially to the institutionalized older individuals which were not involved in this investigation.

In conclusion, it is crucial to highlight the need for future studies investigating not only the impact of the COVID-19 restrictions on the motor and psychological functions in late adulthood but also the short and long-term effects of specific interventions enhancing the psychological and motor health of older people socially and physically deprived because of the COVID-19 outbreak.

## Data Availability

The data that support the findings of this study are not publicly available due to privacy or ethical restrictions.
